# The complete chloroplast genome of *Salix matsudana* f. *tortuosa*

**DOI:** 10.1080/23802359.2022.2110007

**Published:** 2022-10-20

**Authors:** Tiantian Yang, Qiang Zhang, Chengjun Yang, Jian Qiu

**Affiliations:** a Southwest Forestry University, Kunming, PR China; b Northeast Forestry University, Harbin, PR China

**Keywords:** Chloroplast genome, phylogenetic analysis, Salicaceae, *Salix matsudana* f. *tortuosa*

## Abstract

In this study, the complete chloroplast genome of *Salix matsudana* f. *tortuosa* was sequenced and analyzed. The genome of *Salix matsudana* f. *tortuosa* was 155,673 bp in length and was quadripartite in structure, containing a large single-copy region with a length of 84,447 bp, a small single-copy region with a length of 16,320 bp, and two inverted repeats of 27,453 bp in length. The chloroplast genome contains 130 genes, including 85 protein-coding genes, 37 tRNA genes, and eight rRNA genes. The GC content is 36.64%. The phylogenetic tree shows that *Salix matsudana* f. *tortuosa*, *Salix matsudana*, and *Salix babylonica* are closely related and located on the same branch. The chloroplast genome of *Salix matsudana* f. *tortuosa* will provide important data for further systematic study of Salicaceae and the genus *Salix*.

*Salix matsudana* f. *tortuosa* (Vilm.) Rehd. (In 1925) is a member of the Salicaceae family and an arbor willow. In the Chinese flora, it is regarded as a variant of *Salix matsudana.* The main difference from the original variant is that the branches are curled (Wang and Fang 1984). *Salix matsudana* f. *tortuosa* often grows in moist and sandy loam, sometimes on saline soils. *Salix matsudana* f*. tortuosa* is distributed throughout Eastern and Northern China (Liu 2014). It is also a common landscaping tree in Northern China (Zhang et al. 2017).

Fresh young leaves of *Salix matsudana* f. *tortuosa* were harvested from Northeast Forestry University of Harbin, Heilongjiang Province China (45.726582° N, 126.644728° E). The program of plant collection and experiment in the article has passed the ethical review of the Animal and plant Ethics Committee of College of Forestry, Northeast Forestry University. The total genomic DNA was extracted from fresh leaves using CTAB method (Doyle 1987). The DNA library was constructed with insert size of 300 bp fragments and sequencing was performed on the Illumina Novaseq platform based on the Paired-End 150 (PE150) strategy. About 5 Gb raw data were obtained, and the low-quality sequences were filtered by using NGS QC Toolkit_v2.3.3 (Patel and Jain 2012) to produce high-quality clean reads. The chloroplast genome assembly was performed using GetOrganelle (Jin et al. 2020) and annotated using CPGAVAS2 (Shi et al. 2019) and Plastid Genome Annotator (PGA, Qu et al. 2019) software, followed by manual correction via Geneious version 11.0.3 (Kearse et al. 2012) with the genus *Salix* chloroplast genomes (MG262361 and NC_037426) as references. Voucher specimens of *Salix matsudana* f. *tortuosa* were deposited at the Herbarium of Northeast Forestry University, Harbin, China under voucher number Nefu20200720longzhualiu01. All the plant materials and specimens used in the experiment were collected and identified by the author (Chengjun Yang), and the research work was approved by the Ethics Committee of the School of Forestry and the Department of Landscape Planning of Northeast Forestry University.

The chloroplast genome of *Salix matsudana* f. *tortuosa* is 155,673 bp in length, and the GC content is 36.64%. It shows a typical quadripartite structure that includes a large single-copy (LSC) region, a small single-copy (SSC) region, and two inverted repeat regions (IRA/IRB). The length of the LSC region is 84,447 bp, and the GC content is 34.43%. The length of the SSC region is 16,320 bp, and the GC content is 30.99%. The two inverted repeat regions (IRA/IRB) are 27,453 bp, and the GC content is 41.7%. One hundred and thirty genes are encoded in the chloroplast genome, including 85 protein-coding, 37 tRNA, and eight rRNA genes. In addition, two pseudogenes were identified. Among them, *ndh*A, *ndh*B, *pet*B, *pet*D, *atp*F, *rpl*16, *rpl*2, *rpo*C1, *trn*A-UGC, *trn*G-GCC, *trn*l-GAU, *trn*K-UUU, *trn*L-UAA, and *trn*V-UAC contain a single intron, and *rps*12, *clp*P, and *ycf*3 contain two introns. The complete chloroplast genome sequence of *Salix matsudana* f. *tortuosa* has been deposited into GenBank with the accession number MT872638. Comparison of the complete chloroplast genome sequence of *Salix matsudana* f. *tortuosa* to previously published data shows a high level of gene synteny with *Salix matsudana* (NC.059039.1) publicly available on GenBank.

A phylogenetic tree was conducted on the complete chloroplast genome sequences of *Salix matsudana* f. *tortuosa* and 73 other related species (including one outgroup) from the NCBI database and alignments were performed with MAFFT version 7 software (Katoh and Standley 2013). The maximum-likelihood (ML) analysis was performed with RAxML v.8.0 (Stamatakis 2014) using the model of nucleotide substitution to GTR + G and 1000 bootstrap replicates, The method yielded topologies with high support values. The results of the phylogenetic analysis show that *Salix matsudana* f. *tortuosa*, *Salix matsudana*, and *Salix babylonica* are closely related and located on the same branch (Figure 1). The chloroplast genome of *Salix matsudana* f. *tortuosa* will provide important data for further systematic studies of Salicaceae and the genus *Salix.*

**Figure 1.**
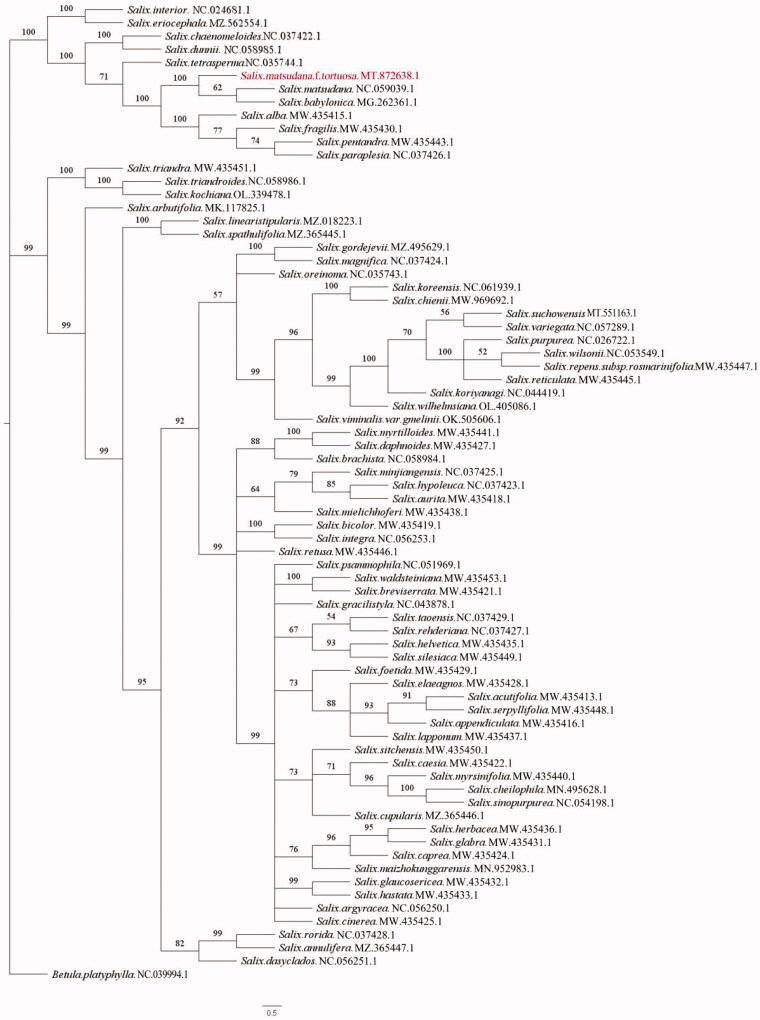
Phylogenetic tree of the genus *Salix* reconstructed using maximum-likelihood (ML) based on the chloroplast genome sequences. Values at the nodes correspond to the support values for ML methods.

## Author contributions

Tiantian Yang, Jian Qiu, and Chengjun Yang conceived and designed the paper; Tiantian Yang drafted the paper; Qiang Zhang analyzed and interpreted of the data; Chengjun Yang collected plant materials, identified specimens, and revised it critically for intellectual content; Jian Qiu contributed reagents/materials/analysis tools, Jian Qiu can provide the final approval of the version to be published. All authors agree to be accountable for all aspects of the work.

## Data Availability

The genome sequence data that support the findings of this study are openly available in the GenBank database of NCBI at https://www.ncbi.nlm.nih.gov/nuccore/MT872638 under accession no. MT872638. The associated BioProject, SRA, and Bio-Sample numbers are PRJNA774821, SRP343274, and SAMN22583583, respectively. The voucher specimens of *Salix matsudana* f. *tortuosa* were deposited at the Herbarium (contact person and email: Chengjun Yang, nxyycj@163.com.) under voucher number Nefu20200720longzhualiu01.
